# Biomechanics of Posterior Dynamic Stabilization Systems

**DOI:** 10.1155/2013/451956

**Published:** 2013-03-31

**Authors:** D. U. Erbulut, I. Zafarparandeh, A. F. Ozer, V. K. Goel

**Affiliations:** ^1^Department of Neurosurgery, School of Medicine, Koc University, Rumelifeneri Yolu, 34450 Istanbul, Turkey; ^2^Department of Mechanical Engineering, Colleges of Engineering, Koc University, Rumelifeneri Yolu, 34450 Istanbul, Turkey; ^3^Departments of Bioengineering and Orthopaedic Surgery, Engineering Center for Orthopaedic Research Excellence (E-CORE), Colleges of Engineering and Medicine, University of Toledo, Toledo, OH 43606, USA

## Abstract

Spinal rigid instrumentations have been used to fuse and stabilize spinal segments as a surgical treatment for various spinal disorders to date. This technology provides immediate stability after surgery until the natural fusion mass develops. At present, rigid fixation is the current gold standard in surgical treatment of chronic back pain spinal disorders. However, such systems have several drawbacks such as higher mechanical stress on the adjacent segment, leading to long-term degenerative changes and hypermobility that often necessitate additional fusion surgery. Dynamic stabilization systems have been suggested to address adjacent segment degeneration, which is considered to be a fusion-associated phenomenon. Dynamic stabilization systems are designed to preserve segmental stability, to keep the treated segment mobile, and to reduce or eliminate degenerative effects on adjacent segments. This paper aimed to describe the biomechanical aspect of dynamic stabilization systems as an alternative treatment to fusion for certain patients.

## 1. Introduction

Lower back pain is one of the major health problems around the world. One of the leading causes of lower back pain is considered to be degeneration of intervertebral disc. Disc herniation, spondylolisthesis, spondylosis, and spinal stenosis may follow intervertebral disc degeneration. Back pain occurs when posterior disc bulges out and impinges the nerve roots due to herniated disc. Another nerve root impingement may be seen in the condition of spinal stenosis, which is a reduction of the diameter of the spinal canal.

The treatment options of lower back pain may vary depending on the severity of the case. They include conservative treatment or surgical techniques. Conservative treatments include exercise, medications, physiotherapy, and rehabilitation. Surgical treatment is considered for the patients when the back pain limits their daily activities and when the condition does not respond to other therapies. Surgical methods include decompression with spinal fusion or nonfusion devices.

Spinal fusion supported by rigid instrumentation is widely used in the treatment of various spinal disorders. Since the procedure was first introduced by Albee and Hibbs in 1911, fusion has played an important role in the lumbar spine employed operations. The ideal result in performing fusion is to gain the necessary therapeutic goals with the minimal disruption of normal structure and function of the spinal column [1, 2]. However, usage of the rigid instrumentation results in a considerable amount of morbidity and of complications. Adjacent disc degeneration is reported by many investigators, known as one of the problems in fusion technique. Omitting the mobility causes the adjacent segments to be overloaded and as a result the number of interventions increases. Considering all these reasons, the search for alternative procedures with different concept was reinforced [3].

In recent years, posterior dynamic stabilization devices have been introduced as a trustworthy alternative to fusion and gained increasing popularity. The comparable advantages of these devices to fusion include retention and protection of the intervertebral disc, earlier surgical intervention, and minimally invasive techniques. Dynamic stabilization technique is aimed at preserving motion at the treated segment. It reduces the risk of accelerated degeneration at adjacent levels, which is a major concern in fusion because of the protective effects of continuing segmental motion [4, 5]. Although dynamic stabilization has gained a lot of attention by the investigators, designing a new spinal-implant system needs a cautious approach. The fusion implant needs to provide the stabilization until the fusion takes place; but for the dynamic stabilization systems, this role should be taken throughout lifetime [6]. So far, various posterior dynamic stabilization systems have been reported in the literature that can be mainly categorized as (1) pedicle screw-based systems and (2) posterior interspinous spacers. In this paper, biomechanical evaluation of posterior spinal implants was described, and biomechanical properties of several such devices were reviewed.

## 2. Biomechanical Evaluation of Dynamic Spinal Implants

Segmental biomechanics will be altered by the implantation. Therefore, it is crucial to evaluate biomechanical effects of implants on the treated and nontreated spinal segments before clinical trials. Biomechanical evaluation of posterior dynamic stabilization systems can be accomplished by *in vivo*, *in vitro*, and finite element analysis (FEA) studies. The change in decompression and stabilization parameters due to instrumentation with respect to intact case can be assessed using spine specimen. It is the so-called *in vitro* studies that include spine specimen from human or other species. *In vitro* studies should follow standard protocols [7] during the preparation of spine specimens and testing. Goel et al. [7] suggested that multispinal segment should be used in order to include one free functional spinal unit (FSU) on each side of the implanted segment. Desired loads are applied to the free end of the specimen, and motion data is recorded accordingly. There are two loading protocols known as displacement control and flexibility control loading. Load control protocol includes force loadings such as shear, pure moment, and complex loads.

**Figure 1:**
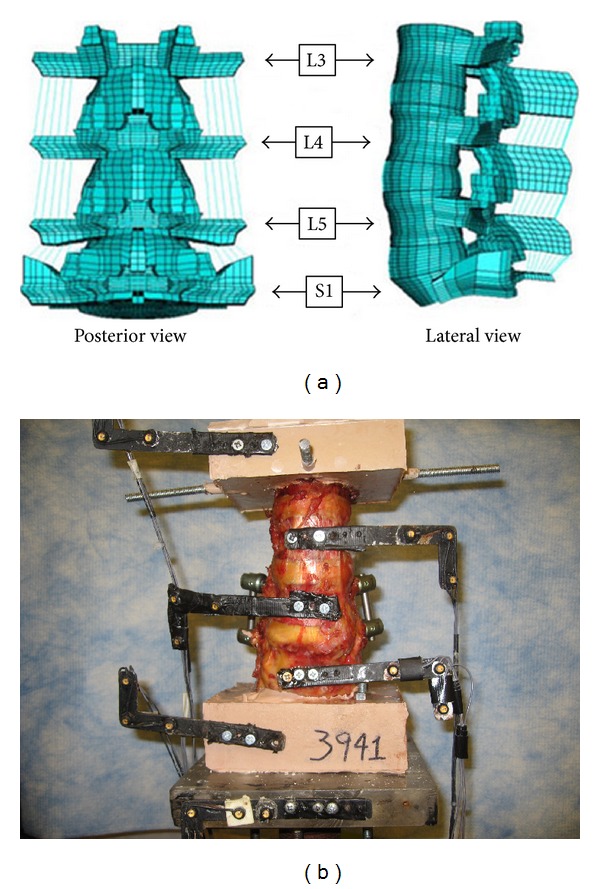
(a) FE model of the lumbar spine (E-CORE, University of Toledo), (b) the lumbar spine specimen with posterior dynamic stabilization system.

FEA plays an important role in biomechanical evaluation of implants. It is helpful to determine the structural analysis of an implant, bone, and interaction in between the two. FE analysis gives full inside of load shearing, stresses, and strain of the interested construct under loading scenarios. It provides prospective outline of needed parameters for a desired spinal implant development. These parameters cannot be determined by *in vitro* experimental studies. However, finite element model needs to be validated by *in vitro* experimental study.  Figure 1 depicts *in vitro* cadaver study and lumbar FE model.

## 3. Posterior Dynamic Stabilization Systems

### 3.1. Pedicle Screw-Based Stabilization Systems

**Figure 2:**
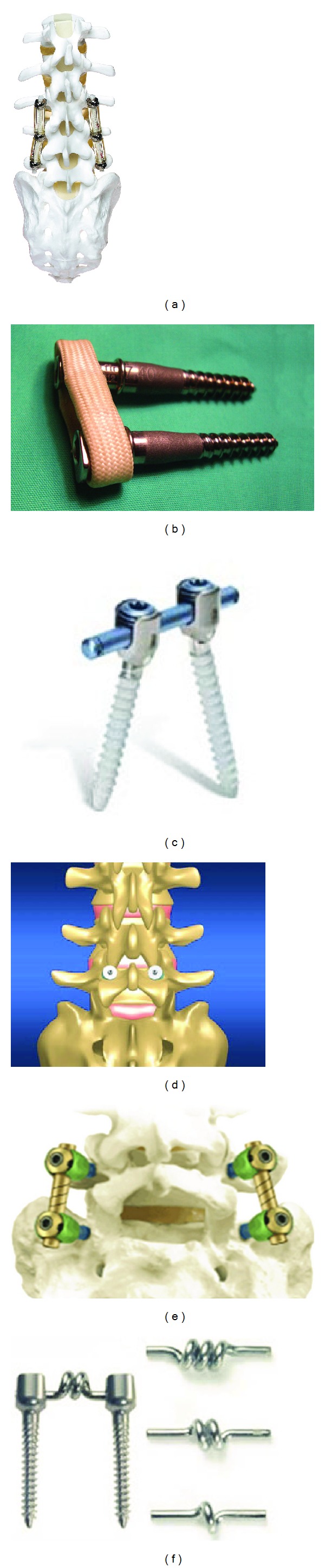
Posterior dynamic stabilization systems. (a) Dynesys; (b) Graf system; (c) PercuDyn; (d) Cosmic; (e) AccuFlex; (f) BioFlex.

The Dynesys (Dynamic Neutralization System for the Spine), known as a dynamic stabilization device, is one of the alternative solutions for the degenerative lumbar disc problems, Figure 2(a). It was implanted for the first time in 1994 by Dubois et al. [8] as a pedicle screw-based system. The intention for using the Dynesys as a flexible posterior spinal fixation system is to maintain intersegmental motions or reduce them to magnitudes found in the intact spine, reducing the negative effects on the adjacent segments. The Dynesys is a bilateral device and consists of titanium alloy pedicle screws and polycarbonate urethane (PCU) spacers that surround tensioned polyethylene terephthalate (PET) cords [9].

Schmoelz et al. [10] performed an *in vitro* study to evaluatethe the biomechanical effect of Dynesys on the magnitudes of stabilization at the treated segment. All the six spines were tested in four stages: the intact, with the defect of the middle segment, fixation with the Dynesys, and fixation with internal fixator. The cadavers were loaded with pure moments in three motion planes, that is, flexion-extension, lateral bending, and axial rotation. The results showed that for the bridged segment, the Dynesys was able to stabilize the spine. The study showed that Dynesys allowed more flexibility to the segment than the internal fixator. In another study with the same loading conditions, Schmoelz et al. [11] investigated the influence of the dynamic stabilization system (Dynesys) on the intervertebral disc which is bridged. It was observed that load bearing of the disc was slightly altered in the case of axial rotation. In flexion, both devices showed a good support of the anterior column by decreasing the intradiscal pressure but slightly below the intact level. Their results showed that the Dynesys did not show substantial differences in intradiscal pressure of the bridged disc compared to the internal fixator. Beastall et al. [12] investigated the biomechanical influence of the Dynesys on the lumbar spine. It was found that Dynesys significantly reduced motion at the bridged segment. However, implantation did not affect the range of motion at the adjacent segment.

Biomechanical investigations reported that some of the posterior dynamic stabilizations show similar effect on flexion, extension, and lateral bending, compared with rigid instrumentation due to dynamic implants with high stiffness [4, 9, 10, 13, 14]. Recent studies suggest that a dynamic implant with lower stiffness may be sufficient to stabilize the spinal segment [13, 15]. Dynamic implants with minimal stiffness of 45 N/mm axially and 30 N/mm bending are enough to reduce spinal flexibility by 30% of the intact range of motion, which is considered to be optimal motion reduction [13]. Another study demonstrated that the optimal axial stiffness value of the longitudinal rods should be approximately 50 N/mm for an effective pedicle screw-based dynamic implant [9]. For example, studies showed that Dynesys (Dynesys-Zimmer, Minneapolis, MN, USA) presents higher stiffness than initially expected [10, 14]. Posterior dynamic fixator with high stiffness does not allow enough mobility to the treated segment in order to have potential benefits as described previously. A dynamic rod with very low axial stiffness (<200 N/mm) did influence the segmental kinematics and allows more mobility [9]. 

Another alternative for the rigid spinal fusion is a soft or flexion stabilization technique introduced by Graf [16]. Graf Ligament (SEM Co., Mountrouge, France) is composed of titanium pedicle screws that are connected by polyester-threaded bands, Figure 2(b). In fact, the polyester bands prevent the abnormal rotary motion and preserve the segment physiological lordosis. Biomechanical investigations have shown that the Graf system reduces the angular motion in flexion extension without limiting the vertebral body translation in other directions. As a result, the Graf system design has drawbacks in preventing the spondylolisthesis. Kanayama et al. [17] studied the efficacy of the Graf system in the treatment of the degenerative spondylolitshesis. Their study included 64 patients that underwent Graf system. Based on the clinical and radiographic results, the vertebral slip could not be prevented, but in 80% of the patients the lordosis was maintained.

Cosmic (Ulrich GmbH & Co. KG, Ulm, Germany) is a posterior dynamic stabilization system using pedicle screws to provide nonrigid stability for the degenerative lumbar spine. The head of the pedicle screws is hinged shaped and it connects the threaded part to the screw. This composition enables the load sharing between the Cosmic and the anterior vertebral column, Figure 2(c). In a study [18], 103 consecutive patients were treated with Cosmic. The results showed a considerable improvement of pain, related stability, and mobility, but 10% reoperation during the followup was observed.

Wilke et al. [15] suggested that if one dynamic system provides 70% less range of motion compared to nondegenerated segment, it may prevent screw loosening. In addition, other studies showed a good agreement that a reduced load in the pedicle screw-based dynamic stabilization system minimizes the risk of screw loosening [19]. However, studies also showed that screw loosening problem can be minimized by using hinged dynamic screws regardless of posterior stabilization systems [20, 21].

The PercuDyn (Interventional Spine Inc., Irvine, CA, USA) is known as an extension-limiting posterior dynamic stabilization implant, which is mainly a bilateral facet augmentation system, Figure 2(d). Two titanium screws anchor the device to the pedicles, and a polycarbonate urethane cushion bumper resting against the inferior auricular process provides the flexibility for the dynamic posterior stabilization system [5, 22]. Masala et al. [23] conducted a study on the PercuDyn implant to evaluate the efficiency of this system as a treatment for patients with lumbar stenosis. The implantation was performed on 24 consecutive patients with lumbar stenosis. The results demonstrated that in 20 patients (83%), 1-year follow-up improvement was observed. For the all patients, including responder and nonresponder, no complications were reported regarding the device.

The Accuflex (Globus Medical, Inc.) is a posterior dynamic stabilization system that achieves the flexibility from helical cuts on rod. It is categorized as a pedicle screw-based system including a dynamic rod and 6.5 mm pedicle screws made of titanium alloy, Figure 2(e). The helical cut transforms the rod into semirigid one that allows motion primarily in the flexion-extension mode. One of the advantages of the Accuflex is that it requires a technique similar to the standard pedicle screw/rod construct and due to that, the insertion can be performed by most spine surgeons [24]. Reyes-Sánchez et al. [25] reported the clinical outcome of a series of patients with lumbar spinal stenosis that underwent lumbar spine instrumentation with the Accuflex implant. Although clinical benefits were observed in 83% of the patients, the failure due to fatigue in 22.22% of the patients led to hardware removal. 

BioFlex, similar to Dynesys, is a posterior dynamic stabilization system using titanium pedicle screws connected by Nitinol rods with coiling consisted of 1-2 turns, Figure 2(f). Nitinol is classified as a shape memory alloy and shows superelasticity behavior. In addition to this property, it is somewhat rigid and it can act as a tension band at the posterior spinal column. The BioFlex system resists excessive deformation during extension; thus, it maintains the physiological range of motion [26].

### 3.2. Interspinous Spacers

**Figure 3:**
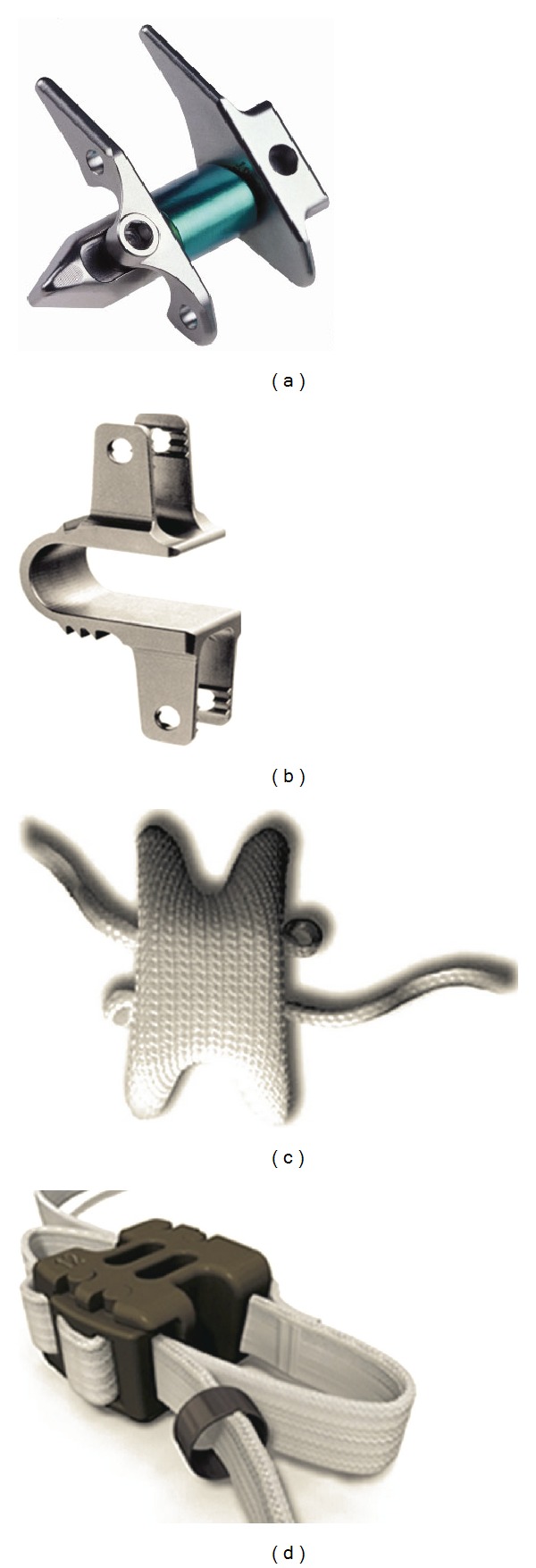
Interspinous spacer: (a) X-STOP, (b) Coflex, (c) DIAM system, and (d) Wallis system.

The lumbar interspinous process decompression (IPD) devices are known as trustable alternatives for the treatment of various spinal disorders. The first IPD device, X-STOP (St Francis Medical Technologies, Alameda, CA, USA), was introduced in the US for the treatment of patients with neurogenic intermittent claudication due to spinal stenosis, Figure 3(a). The major concept in the design of X-STOP is to limit the extension movement at the individual stenotic level while allowing the normal movement in all other directions of the treated and untreated level(s) [27]. Compared to other IPD devices, X-STOP has been documented extensively in the literature. Its features include two lateral wings to prevent migration, intended to distract the discs, increase the foraminal areas, and stabilize the posterior column [5]. Lindsey et al. [28] studied the effect of X-STOP device on the kinematics of the instrumented and adjacent levels. They tested seven lumbar spines (L2–L5) in three motion planes: flexion-extension, lateral bending, and axial rotation. The study showed that X-STOP did not affect the kinematics of the adjacent segment. Siddiqui et al. [29] studied kinematics of the lumbar spine with X-STOP device in sagittal plane at the instrumented and adjacent levels *in vivo*. They measured the disc heights, endplate angles, and segmental and lumbar range of movement after implanting the X-STOP. The results showed that no significant changes were seen in disc heights and segmental and total lumbar spine movements postoperatively. They concluded that the sagittal kinematics of the lumbar spine is affected using X-STOP. Kondrashov et al. [27] performed a 4-year followup on 18 X-STOP subjects. Twelve patients had the X-STOP implanted at either L3-4 or L4-5 levels while the other 6 patients had the X-STOP implanted at both L3-4 and L4-5 levels. Grade *Ι* spondylolisthesis was noticed in six patients.

Coflex device (Paradigm Spine, LCC, New York City, NY, USA), formerly Interspinous “U,” is one of the dynamic interspinous implants that was first introduced by the French orthopaedic surgeon Jacques Samani as an alternative to arthrodesis, Figure 3(b). The aim of this U-shaped compressible device, which is manufactured from titanium, is to unload the facet joints, restore the foraminal height, and provide stability in order to improve the clinical outcome of surgery [30]. 

Diam implant (Medtronic, Memphis, TN, USA) system is an interspinous spacer and it has a silicon core with a polyethylene cover [31]. Three mesh bands are designed to secure the implant: two of them are around each spinous process and the other around the supraspinous ligament, Figure 3(c). 

In mid-1980s, Sénégas introduced an interspinous implant, which was a “floating system” with the purpose of avoiding the risk of loosening. The implant system consisted of a titanium spacer placed between the spinous processes of the lumbar spine. Two Dacron ligaments wrapping around the spinous processes were considered to secure the implant. Despite the favorable results, a second-generation device called Wallis (Spine Next, Bordeaux, France) was developed to improve the device functionality (Figure 3(d)). In the newer implant, the polyetheretherketone (PEEK) is replaced with the titanium. Sénégas recommends that the current design of implant can be used for lumbar disc disease in the following indications: (1) discectomy for a herniated disc with a large material loss, (2) a second discectomy for recurrence of herniated disc, (3) discectomy for herniation of a transitional disc with sacralization of L5, (4) degenerative disc disease at a level adjacent to a previous, and (5) isolated Modic I lesion leading to chronic lower back pain [32]. 

## 4. Conclusions 

Fusion is a gold standard for lower back pain treatment to date. However, there have been several complications reported clinically. These complications are related mainly to adjacent segment degeneration due to high stiffness at the stabilized segment. Alternative treatment, nonfusion stabilization systems, became more and more popular in order to preserve mobility of a motion segment and eliminate adjacent segment phenomena. Current research studies emphasize long-term clinical evaluation of dynamic stabilization system. 

On the other hand, the stiffness of the dynamic implants is a big concern due to not providing appropriate motion range. Therefore, it is important to optimize the dynamic implant stiffness for desired spinal range of motion achievement.
